# The Early Transmission Dynamics of H1N1pdm Influenza in the United Kingdom

**DOI:** 10.1371/currents.RRN1130

**Published:** 2010-06-13

**Authors:** Azra Ghani, Marc Baguelin, Jamie Griffin, Stefan Flasche, Albert Jan van Hoek, Simon Cauchemez, Christl Donnelly, Chris Robertson, Michael White, James Truscott, Christophe Fraser, Tini Garske, Peter White, Steve Leach, Ian Hall, Helen Jenkins, Neil Ferguson, Ben Cooper

**Affiliations:** ^*^MRC Centre for Outbreak Analysis & Modelling, Imperial College London; ^†^Health Protection Agency, London, UK; ^‡^Health Protection Agency, London, UK / University of Strathclyde, Glasgow, UK; ^§^Health Protection Agency; ^¶^MRC Centre for Outbreak Analysis and Modelling, Department of Infectious Disease Epidemiology, Imperial College London; ^#^MRC Centre for Outbreak Analysis and Modelling, Imperial College London; ^**^Strathclyde University; ^††^MRC Centre for Outbreak Analysis and Modelling, Dept. of Infectious Disease Epidemiology, Imperial College London; ^‡‡^Imperial College London; ^##^HPA Emergency Response Department, Porton Down; ^***^Imperial College and ^†††^Director, MRC Centre for Outbreak Analysis and Modelling, Imperial College London

## Authors

Azra C Ghani^1^*, Marc Baguelin^2^*, Jamie T Griffin^1^*, Stefan Flasche^2,6^*, Richard Pebody^2^*, Albert Jan Van Hoek^2*^, Simon Cauchemez^1^, Ian M Hall ^3^, Christl A Donnelly^1^, Chris Robertson^4,6^, Michael T White^1^, Iain Barrass^3^, Christophe Fraser^1^,  Alison Bermingham^2^, James Truscott^1^, Joanna Ellis^2^, Helen E Jenkins^1^, George Kafatos^2^, Tini Garske^1^,  Ross Harris^2^, Jim McMenamin^4^, Colin Hawkins^2^, Nick Phin^2^, André Charlett^2^, Maria Zambon^2^, W. John Edmunds^2,5^,  Mike Catchpole^2^, Steve Leach^3^, Peter J White^1,2^, Neil M Ferguson^1+^, Ben S. Cooper^2+^.  

 1. MRC Centre for Outbreak Analysis & Modelling, Department of Infectious Disease Epidemiology, Imperial College London

2. Centre for Infections, Health Protection Agency

3. Emergency Response Department, LaRS Porton Down, Health Protection Agency

4. Health Protection Scotland

5. London School of Hygiene and Tropical Medicine 

6. University of Strathclyde

 *These authors contributed equally. + Correspondence to neil.ferguson@imperial.ac.uk or ben@tropmedres.ac.

## Abstract

 We analyzed data on all laboratory-confirmed cases of H1N1pdm influenza in the UK to 10th June 2009 to estimate epidemiological characteristics. We estimated a mean incubation period of 2.05 days and serial interval of 2.5 days with infectivity peaking close to onset of symptoms. Transmission was initially sporadic but increased from mid-May in England and from early June in Scotland. We estimated 37% of transmission occurred in schools, 24% in households, 28% through travel abroad and the remainder in the wider community. Children under 16 were more susceptible to infection in the household (adjusted OR 5.80, 95% CI 2.99-11.82). Treatment with oseltamivir plus widespread use of prophylaxis significantly reduced transmission (estimated reduction 16%).  Households not receiving oseltamivir within 3 days of symptom onset in the index case had significantly increased secondary attack rates (adjusted OR 3.42, 95% CI 1.51-8.55).

## Introduction

H1N1pdm influenza, which first emerged in Mexico in March 2009, has now spread rapidly across the globe. At the start, this new virus prompted questions about how to best use limited resources to mitigate the effects of the pandemic. Pre-pandemic modeling work suggested that layered mitigation policies, including the use of antivirals and social distancing (such as school closure) could have an important impact on overall and peak clinical attack rates [1] [2] [3] [4]. These assessments of intervention strategies, however, depended critically on the transmission potential, natural history of infection, antiviral effectiveness and age-related patterns of susceptibility for the H1N1 pandemic strain. 

Early estimates of these quantities for the new virus using data from Mexico suggested that the reproduction number, R, was slightly lower than estimated in previous pandemics, with estimates in the range 1.4-1.5 [5] [6] [7]. These data also showed higher attack rates in children compared to adults, although this was based on observations in a single village and virological confirmation was lacking [5]. Early surveillance relying on active case finding highlighted the importance of transmission between children in schools in the early phases of the pandemic in many countries [8] [9] [10] [11] [12]. This may in part have been due to prior cross-protective immunity in adults, as demonstrated by a serological study [13]. However, differences in patterns of mixing between adults and children may also have in part determined these apparent differences. Thus detailed epidemiological analysis of case data that take into account the place of contact and age-related heterogeneities in mixing patterns was required to assess the degree of susceptibility compared to the effect of social mixing patterns. These parameters are key to assessing the potential impact of school closures and other social distancing measures on the speed and overall attack rate of autumn waves of the pandemic in the Northern Hemisphere. 

 As part of the pandemic planning process in the UK, the use of mathematical models in real-time was conceived to be part of the overall response and to provide direct advice to government.  To enable this, data collection studies were also planned, designed to collect detailed epidemiological data at an individual level for the first few hundred cases.Here we present a comprehensive quantitative analysis of these early data collected as part of the epidemiological investigation of laboratory-confirmed cases of H1N1pdm influenza in the UK up to June 10^th^. This analysis was undertaken in real-time, providing regular up-to-date advice to the UK government during the early phase of the pandemic on the transmission potential, attack rates and effectiveness of the containment policy (see UK Department of Health Planning Assumptions). From a scientific perspective, these data uniquely provided an opportunity to estimate key transmission parameters that can no longer be obtained given the shift away from individual case reporting. Because detailed contact tracing of all cases was undertaken, they can be used to directly estimate the incubation period and serial interval rather than making indirect inferences from case report data or from individual settings [7]. A particular focus of the UK’s containment strategy was wide-scale use of oseltamivir both for treating cases and prophylaxis of their close contacts. By combining data on timings of treatment and prophylaxis with our estimates of susceptibility and onward transmission we were therefore able to explore the impact that antiviral use had on onward transmission in the first two months of the pandemic. 

## Methods

### Data Sources

We analysed data on all laboratory-confirmed cases from the United Kingdom , including age, gender and postcode of the case, dates of symptom onset and of laboratory confirmation [14] [15]. Recent travel abroad and the date of return to the UK, known epidemiological links to other cases, the school attended  and antiviral treatment were also recorded. The First Few Hundred Cases (FF100) study collected more detailed data on a subset of cases including household contacts. Household size was assumed to be the sum of all reported household contacts plus the index case.  Further details of these datasets are presented elsewhere [11] [16]. We analysed data for the entire UK and in two strata: England/Wales (also including a very small number of cases from Northern Ireland) and Scotland.

###  Source of Infection & Cluster Definitions

Information on known epidemiological links to other cases (household, school and reported contact data) was used to partition the data into clusters. A cluster was defined as the set of cases with known epidemiological links to each other within the UK.  The root of each cluster’s infection tree, the cluster index case, was therefore either believed to have been infected abroad or had no known epidemiological links to earlier cases. As some individuals had more than one potential source of infection, we also considered an alternative approach in which the probability of infection from each potential source was determined probabilistically using the estimates of the incubation period and infectivity distribution (see below and Technical  Appendix).

### Incubation Period and Infectivity Distributions

The incubation period distribution was estimated by maximum-likelihood fitting of a Gamma distribution to the 16 cases linked to a single exposure.  Data on 60 cases who reported travel abroad, had dates of travel recorded and an onset of symptoms after their return were used to estimate a minimum incubation period (return date to onset date). An individual’s infectivity profile was obtained by fitting a model (see technical appendix) to the serial interval data (onset of symptoms in index case to onset of symptoms in contact) obtained from 58 individuals with a unique source of transmission given the estimated incubation period distribution. 

### Transmission Models & Age-Dependency in Transmission Rates

We estimated the effective reproduction number R from UK population-level data (i) using the observed rate of exponential growth and serial interval distribution [17], with  a change-point analysis to test for changes in the rate of epidemic growth; (ii) by analysing the distribution of the cluster sizes, accounting for censoring due to ongoing transmission within clusters [18]; (iii) by probabilistically reconstructing the epidemic tree using only onset times and the serial interval distribution and adjusting for censoring [19] [20]. These population-level estimates mask considerable heterogeneity in transmission occurring in the early stages of the UK epidemic. We therefore developed 3 new inference methods which incorporate detailed epidemiological information on the patterns of contact, allowing a more accurate assessment of who infected whom. The first two methods extend the tree-reconstruction method [19] [20]. In the first we use the household data and imputed cluster identifiers to reconstruct the transmission network based on the observed serial interval distribution (Model 1). In the second we incorporate contact information as derived probabilistically above based on the estimated incubation period and infectivity distributions (Model 2). The third uses the same contact information as the second but fits a full epidemic transmission model using a renewal equation approach and Bayesian MCMC methods to infer infection times and to estimate model parameters, including the reproduction number over time (Model 3).  Full details for each approach are given in the Technical Appendix. Patterns of age-dependent susceptibility to infection were estimated using data on the approximate numbers  of travelers to the USA and Mexico in different age groups obtained from the International Passenger Survey [21]. Assuming the same exposure to infection in individuals travelling to these countries, estimates of age-dependent susceptibility to infection were derived from the observed patterns of importation of cases from those countries.

### Household Studies

Household secondary attack rates were calculated for cases in the FF100 study. The household index case was defined as the case with earliest onset .Households with co-primary cases were excluded from the analysis. Secondary cases were defined as (a) H1N1 laboratory-confirmed infection or (b) exhibiting clinically defined ILI using CDC definitions (fever plus one or more of cough and sore throat). Logistic regression was used to assess the dependence of household secondary attack rates on household size, age of the index case, age of the secondary contact, timing of treatment in the index case and treatment/prophylaxis of household members.

### Treatment

Using Model 3, we fitted an additional parameter in which the hazard of transmission was reduced from the day after treatment started to the end of the infection. This fitting was only undertaken in the subset of data from England/Wales with missing treatment data considered as additional nuisance parameters within the MCMC fitting algorithm. 

## Results

### Data Summary

The first case of H1N1pdm was confirmed in the UK on 27^th^ April 2009 in an individual returning from Mexico. 817 virologically-confirmed H1N1 cases were reported to June 10^th^ 2009. An additional 192 cases were reported after that date but had recorded onset of symptoms prior to June 10^th^ 2009. Of the 817 cases, 490 had at least one documented potential source of infection. Of these 155 (32%) reported recent travel abroad, 169 (34%) reported household contact with a confirmed case, 198 (40%) had attended an affected school and 69 (14%) reported contact with a confirmed case outside school and household.  295 had associated treatment data recorded, with the majority of these being cases that occurred in England or Wales.

Contact data was collected for a subset of 355 cases who completed the FF100 study questionnaire. Of these, following various exclusions (see technical appendix), the household analysis was based on 193 households (and hence index cases) and 556 secondary household contacts.

### Natural History 

Using data from those that travelled abroad we estimate a minimum incubation period of 1.9 days from their dates of return travel to the UK.  Using data from a single exposure event (bus travel to a football match on 24^th^ May 2009) with 16 secondary cases we estimate an incubation period distribution with mean 2.05 days and variance 0.24 days^2^ (Figure 1B), consistent with values for seasonal influenza A virus (23) . Using these estimates, we obtain an infectivity distribution in which infectivity peaks close to the onset of symptoms (Figure 1D) (mean time from symptom onset to peak infectivity 0.45 days) with a good fit to the observed serial interval distribution (Figure 1C). The true serial interval distribution, allowing for the possibility of tertiary transmissions amongst the observed distribution, was estimated to have a mean of 2.51 days and standard deviation of 1.55 days.

**Figure:**
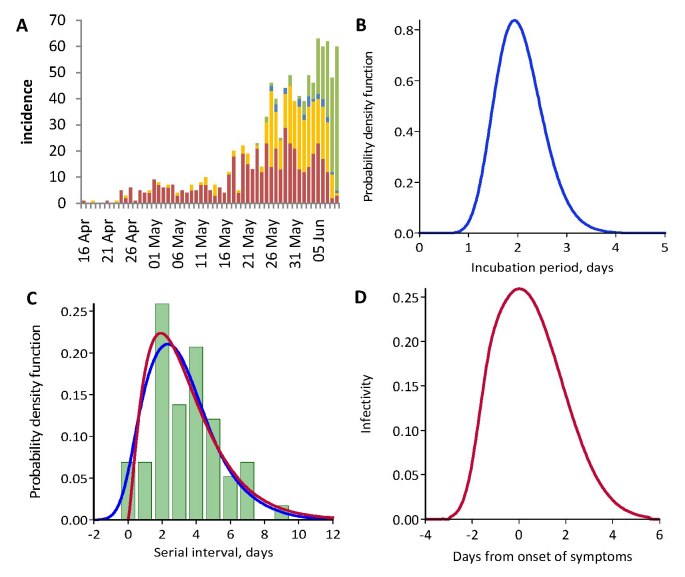
**Figure 1:** A) Time series of 817 confirmed H1N1 cases in England/Wales (red) and Scotland (orange) by date of onset of symptoms with sampled missing onset dates in blue. Additional cases reported after 10th June but with onsets prior to 10th June are shown in green. B) Fitted incubation period distribution (mean 2.05 days and variance 0.24 days^2^).  C) Empirical serial interval distribution (green). The blue line shows the fit of the model allowing for tertiary transmission and the red line the estimated serial interval distribution. D) Estimated infectivity of index cases in relation to their onset of symptoms. The graph shows the probability density function of estimated infectivity profile after integrating out the index cases’s infection time (see technical appendix).

### Source of Infection

For the 60% of confirmed cases that reported at least one documented potential source of infection, using a method in which multiple recorded sources of infection are assigned a probability based on the timing of their infection (see Technical Appendix), we estimated amongst those cases that reported at least one source of infection that 28% (95% CI: 26-34%) were infected abroad (in either Mexico or United States), 37% (95% CI: 33-42%) through contacts made at school and 24% (95% CI: 18-26%) within the household, with the remainder of transmission occurring outside the household or school locations (including other relatives, workplace and social contacts). Cases were geographically dispersed, but with central foci around London, Birmingham and Glasgow. 40% of cases had no clear epidemiological links to other cases but we cannot exclude the possibility that these cases acquired their infection in any of the settings noted above.   

A key characteristic of the England/Wales data was the disproportionate number of cases in children, driven primarily by school outbreaks (Figure 2A). In contrast, outbreak data from Scotland showed less clustering, with household transmission playing a greater role (Figure 2B). Also notable is that the England/Wales data contained many more cases where infection was most probably acquired abroad (36%, 95% CI 31-41%) compared to Scottish data (15%, 95% CI 7-18%). The extent to which these differences reflect real epidemiological variation or differences in surveillance is unclear.

**Figure:**
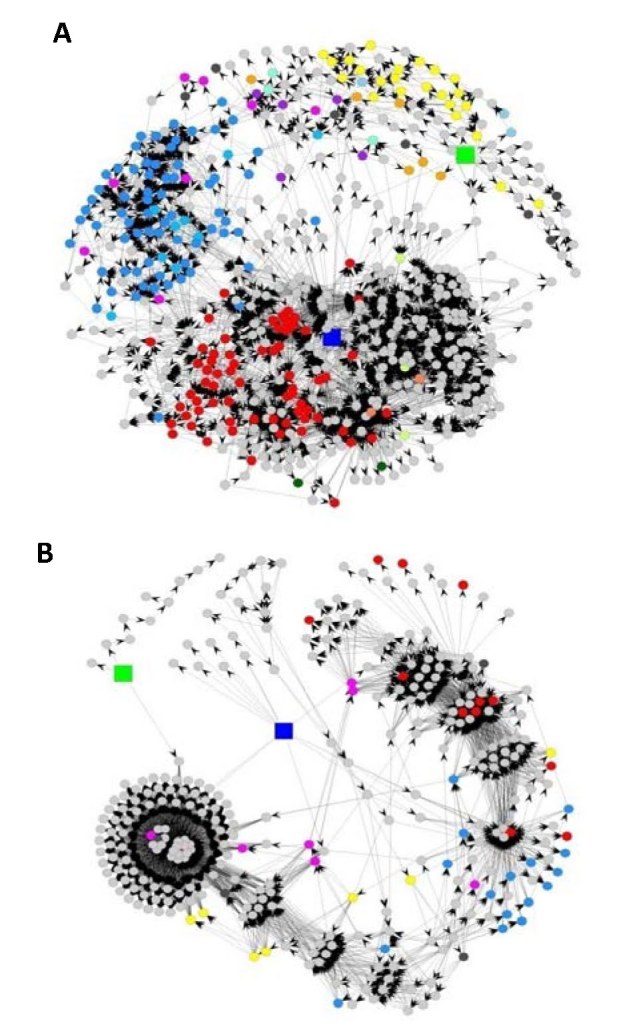

**Figure 2:** Most likely transmission network obtained using Model 2. Circles represent individuals and lines denote paths of transmission. The large green and blue squares represent infection abroad in Mexico and United States respectively. Colours of smaller circles represent the school attended, school contacts or other location contacts. A) England/Wales and B) Scotland. 

### Reproduction Number Estimates Over Time

A change-point analysis revealed strong evidence that the epidemic did not grow at a constant rate (p=0.010, Davies test) and estimated that the growth rate in the UK as a whole changed close to May 15^th^ (95% CI May 8^th^ - May 21^th^) (Figure 3A). Based on the rate of exponential growth before and after this date, estimated values for *R* were 1.06 (95% CI 0.93-1.19) and 1.44 (95% CI 1.27-1.63), respectively. Analysis of the distribution of  cluster sizes using UK data up to May 15^th^ gave a similar estimate for the initial R value ( 1.12 [95% CI  0.79 – 1.64]).

Using both tree-reconstruction methods and fitting a full transmission model gives greater insight into the changes that occurred in the different regions. In both England/Wales and Scotland *R* stayed close to 1 (the threshold value below which transmission cannot be sustained) for the first few weeks of the epidemic (Figure 3B,C). However, from the middle of May onwards, *R* estimates rose above 1 for a short period of time although they dropped shortly after that in England (Figure 3B/C). These early estimates were substantially driven by the detection of a small number of relatively large school-based outbreaks. From early June sustained transmission was clearly underway in both England/Wales and Scotland(Figure 3B,C) with *R* estimates in this final period (31^st^ May to 7^th^ June) in the range 1.2 to 1.5 (England/Wales Model 1: *R*=1.42 (95% CI 1.29-1.57); Model 2: *R*=1.20 (95% CI 1.00-1.41); Model 3: *R*=1.31 (95% CI 1.09-1.56); Scotland Model 1: *R*=1.13 (95% CI 1.03-1.19); Model 2: *R*=1.41 (95% CI 1.02-2.13); Model 3: *R*=1.48 (95% CI 1.28-1.70)).  

**Figure:**
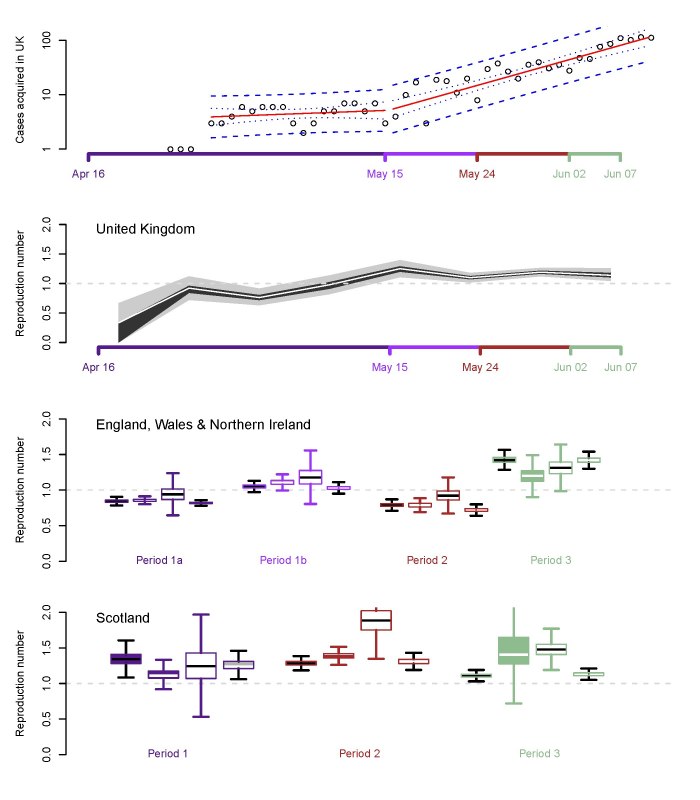

**Figure 3:** A) Fit of exponential growth model to the time series of onsets. The red curve shows the best fitting model in which the change in the exponential growth rate occurs from May 15^th^ onwards (95% confidence bounds and prediction bounds are shown by dotted and dashed lines respectively). Circles denote the observed numbers of onsets excluding cases with a reported travel history. B) Estimates of the weekly population-level reproduction number over time. The white line shows the mean, black shading the interquartile range and grey shading the 95% range. C)-D) Estimates of the effective reproduction number over different time periods calculated using four different models C) England/Wales/Northern Ireland and D) Scotland. Box-plots show R estimates for each time period. Period 1 comprises 1a (cases to May 14^th^) and 1b (May 15^th^-May22nd), period 2 is May 23^rd^-May30th, and period 3 is 1^st^ May-7^th^ June.  For each period the four box-plots represent (from left to right) models 1 to 3 and model 4 (population-level reproduction number). 

### Age Heterogeneity

Analysis of 193 households gave a 5.5-fold higher secondary attack rate for confirmed H1N1pdm and 3.6-fold higher secondary attack rate for ILI in child secondary contacts compared with adults (Table 1).  

**Table 1: **Univariate attack rates, adjusted odds ratios and 95% confidence intervals for the probability of a secondary case in a household with an index confirmed H1N1 case by index age, contact age, household size and the time from the index onset of symptoms to receipt of prophylaxis in the contacts. Based on analysis of 556 household contacts of index cases in 193 households in whom the overall secondary attack rate for virologically-confirmed A/H1N1 is 8.1% and ILI is 11.2%. 

**Table:** 

	Virologically-confirmed A/H1N1			ILI		
Covariate	Attack Rate	Adjusted Odds Ratio (95% CI)	p-value	Attack Rate	Adjusted Odds Ratio (95% CI)	p-value
Age of the contact:						
16 years and under	31/179 (17.3%)	5.80 (2.99,11.82)	<0.001	34/179 (20.7%)	3.77 (2.17, 6.68)	<0.0001
over 16 years	14/377 (3.7%)	1.0	-	25/377 (6.6%)	1.0	-
Age of the index case:						
16 years and under	31/345 (9.0%)	1.14 (0.57, 2.35)	0.78	38/345 (11.0%)	0.76 (0.43, 1.36)	0.34
over 16 years	14/211 (6.6%)	1.0	-	24/211 (11.4%)	1.0	-
Household size	-	1.00 (0.85, 1.16)	0.62	-	1.05 (0.92, 1.19)	0.42
Rapid provision of oseltamivir in the household						
Yes	8/165 (4.5%)	1.0	-	19/165 (11.5%)	1.0	-
No	24/186 (12.9%)	3.42 (1.51, 8.55)	0.005	28/186 (15.0%)	1.45 (0.77, 2.81)	0.26
Not recorded	13/205 (6.3%)	1.41 (0.57, 3.74)	0.46	15/205 (7.3%)	0.59 (0.28, 1.22)	0.16

*Timing of receipt of oseltamivir in household contacts is highly correlated with the timing of receipt of treatment in the index case and therefore these two effects cannot be distinguished in this analysis. 

Furthermore, analysis of the age distribution of cases returning from travel to Mexico or the US, adjusted by age-stratified probabilities of travel to these locations, gave higher risk of infection in children compared to adults, consistent with a higher susceptibility (Figure 4).

**Figure:**
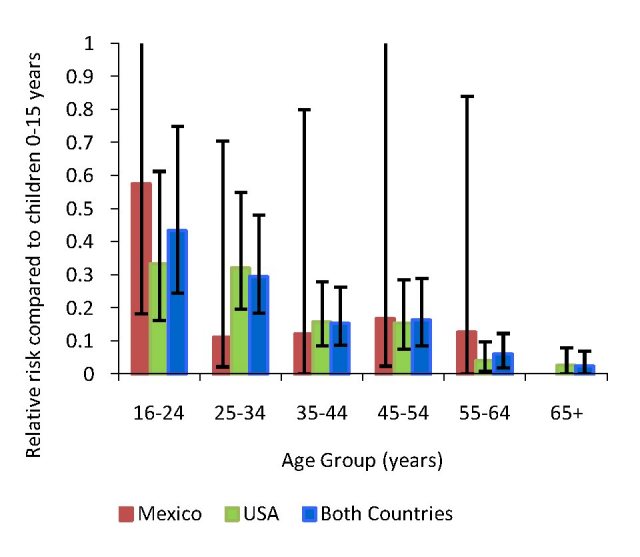
**Figure 4:** Estimates of the age-specific relative risk of infection abroad obtained by analyzing cases with reported travel history and age-specific information on travel to affected countries from the International Passenger Survey (22) .

### Treatment and Prophylaxis

Of a subset of 295 cases for whom treatment data was available, 271 (94%) reported receiving oseltamivir as treatment with a median time from onset of symptoms of 3 days (interquartile range 1 to 5 days) (Figure 5A).  93% of their household contacts reported receiving oseltamivir with a median time from onset of symptoms in the index case of 4 days (Figure 5B). The majority of these contacts received oseltamivir as prophylaxis (defined as receipt prior to the onset of symptoms if a secondary case or at any time period if a negative contact). Fitting the full transmission model allowing a reduction in transmission starting one day after treatment started, we found that treatment (in association with prophylaxis given to contacts) significantly reduced the hazard of infection (estimate 94%, 95% CI 72%-100%). However cases were treated (and their contacts given prophylaxis) several days into their infection when we estimate their onward infectiousness had already started to decline. Hence the overall impact of treatment and prophylaxis needs to take into account these timings. Attributing all the reduction to a direct effect of case treatment and using the estimated infectivity profile and the timing at which treatment was received we estimate that, at a population-level, treatment of cases in association with prophylaxis of their contacts reduced overall transmission by 16% (95% CI 12-20%). As a large proportion of this effect may be due to widespread prophylaxis in close contacts, the overall impact of a treatment-only policy may be substantially less. Overall, our analysis suggests prompter treatment *and* prophylaxis could substantially reduce *R*, assuming all cases are detected (Figure 5C). We also found a significant impact of timing of first receipt of oseltamivir by secondary contacts within the household on the risk of confirmed infection, with members of households which received oseltamivir more than 3 days after the onset of symptoms in the index case significantly more likely to be infected compared to members of households that received oseltamivir after this time (adjusted odds ratio 3.42, 95% CI 1.51-8.55) (Table 1). Whilst there was also an impact of rapid receipt of oseltamivir on ILI, this result was not statistically significant (adjusted odds ratio 1.45, 95% CI 0.77-2.81). Those for whom data on the date of first receipt in the household was not available were also at higher risk of confirmed infection although this result was not statistically significant. Those with no recorded treatment times were less likely to have ILI than other groups;  however this is likely due to a lack of recording of symptoms in these contacts.

**Figure:**
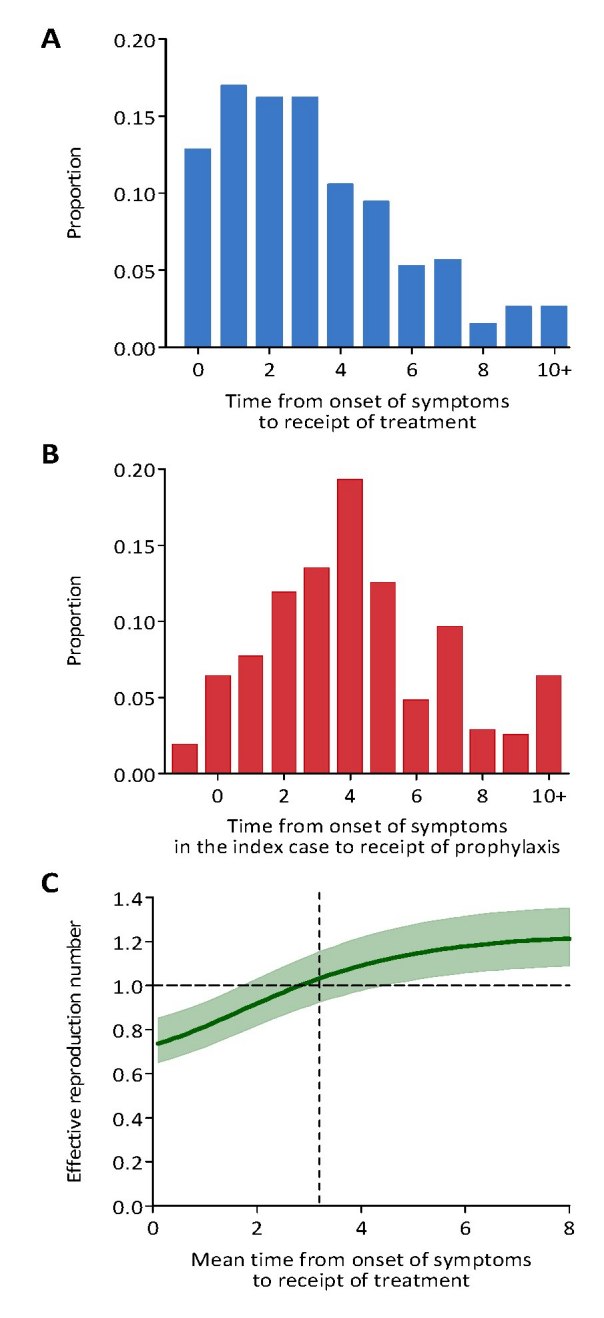

**Figure 5: **A) Distribution of time from onset of symptoms to receiving treatment with oseltamivir in cases. B) Distribution of the time from onset of symptoms in the index case to receiving prophylaxis in 343 contacts of household index cases. C) Estimated relationship between the time from onset of symptoms to receiving treatment for confirmed cases and the effective reproduction number.  The vertical dotted line shows the mean time observed in the cases. As a proportion of infectivity occurs prior to the onset of symptoms (Figure 1D) treatment cannot fully block transmission. 

## Discussion 

From the first UK H1N1pdm cases in April 2009, transmission in the early weeks remained sporadic with early containment measures, including the use of antivirals and reactive school closures, reducing the initial rate of growth of the pandemic. Thus during this early phase our results indicated that the containment policy was effective. However from mid-May sustained transmission was underway in different areas of the country.  From the end of May, infection spread more widely with values for *R *during this final period (range 1.4 to 1.6) consistent with studies elsewhere [5] [6] [7], demonstrating that containment was no longer possible. This new H1N1 virus therefore appears to be less easily transmitted than previous strains. Thus second waves of infection now underway are likely to evolve more slowly and have lower peak attack rates than predicted by pre-pandemic models.

Our results demonstrate substantial age-related heterogeneity in transmission, with children playing a key role via school-based outbreaks. Age-related case ascertainment bias could account for some of this observation. However, two pieces of evidence demonstrate increased susceptibility amongst children. First, analysis of secondary household attack rates indicated significantly higher susceptibility to infection in children compared to adults. Second, analyses of age-stratified infection rates among travellers to Mexico and the US also suggest higher susceptibility in children, consistent with a larger household-based study of H1N1pdm in the United States [22]. These differences in susceptibility are likely due to partial immunity in adults, as shown by higher levels of cross-reactive antibodies in serological studies undertaken in the US [13]. Further serological surveys are required to more fully interpret these age-patterns.

Using detailed contact tracing data we were able to directly estimate  a mean incubation period of 2.05 days, consistent with estimates from seasonal influenza [23], and a mean serial interval of 2.5 days. By combining these estimates it is clear that onward infectivity peaks close to the onset of symptoms, and thus that a substantial amount of transmission occurs prior to the onset of symptoms.  We estimated a significant reduction in infectiousness from the time treatment/prophylaxis was received. However, it is not possible to estimate to what extent this was due to treatment of the index cases or due to the effectiveness of prophylaxis in preventing infection, due to the widespread provision of both during this containment phase. Thus the overall impact of a treatment-only policy on transmission may be substantially less than our 16% estimate, as suggested by prior modeling [3]. In addition, if only a proportion of all cases were ascertained in the early epidemic (as seems probable) the net effect of antiviral use on the epidemic will have been even smaller. We also found a significant impact of rapid provision of oseltamivir to household contacts, with a 71% reduction in laboratory-confirmed H1N1pdm influenza and 31% reduction in symptom-based ILI in those contacts whose household received oseltamivir within 3 days of onset of symptoms in the index case compared with members of households who received oseltamivir later than this. Whilst this effect is robust (see sensitivity analyses in Technical Appendix), its magnitude is uncertain due to the small numbers of secondary cases. Based on previous studies [24] [25] [26] it is biologically more plausible that the protective effect observed was largely due to prophylaxis. An important caveat is that 44% of household contacts were missing information on the time of antiviral use and substantial bias can therefore not be precluded.

One limitation of our analyses is that they rely on laboratory-confirmed cases. One surveillance scheme run in sentinel primary care settings identified 6 cases during the first month of the pandemic. This survey covers a population of approximately 400,000 individuals giving an incidence of 1.5 per 100,000 population per month. Thus in a population of 61 million people we would have expected 915 cases (95% CI 183 - 1646) in total to have sought medical attention, higher than the 253 reported by this date. Thus it may be that the confirmed cases represent a small proportion of all early cases. In a similar scheme in Scotland where the coverage is approximately 100,000 patients in 20 GP practices,  no cases were identified. The surveillance protocol used involved primarily testing clinical cases of ILI if they had an epidemiological link to other confirmed cases or an H1N1pdm-affected country. As the outbreak progressed, clinical surveillance also identified several large school-based outbreaks the scale of which prompted testing even in the absence of epidemiological linkage. Thus, the active case finding-based surveillance approach adopted may have preferentially identified large outbreaks in schools over smaller clusters of cases in the wider community, leading to potential case ascertainment biased. 

Following the shift to ILI-based reporting for H1N1 from July onwards, sustained transmission of H1N1pdm influenza continued in the UK over the summer months, albeit at a lower rate. The central role that children and schools played in the early phase of the pandemic, coupled with the observed reduction in transmission over the summer indicate that the normal closure of schools over the summer holiday period may have reduced transmission. The characteristics of the early phase of the pandemic, with milder illness occurring predominantly in children, is consistent with milder pandemics (such as 1957) and other epidemics such as the outbreak of H1N1 swine influenza in the United States in 1976 [27]. This is further supported by the pattern of cases and limited severe disease in the Southern Hemisphere indicating a relatively mild virus. However, continued careful epidemiological and virological monitoring of the transmission characteristics of the virus over the UK influenza season is required to detect any changes in virulence. 

## Acknowledgements

At the HPA we thank Colin Campbell and Edward Wynne-Evans for data collection and management, Julie Robotham and Elisabeth Adams for co-ordination activities, Sam Bracebridge for support and data collection, Angie Lackenby for technical oversight of performance of virology assays for diagnosis of infection, Phil Sansom for his help in the use of the IPS data, Nick Andrews for helpful discussions, John Watson, Jon Green, Mary Chamberland and Nigel Gay for key roles in developing the FF100 and helpful comments. We are particularly indebted  to staff at HPA LaRS, Health Protection Scotland, Communicable Disease Surveillance Centre Northern Ireland, National Public Health Service Wales, and the Regional Microbiology Network  who participated in FF100 protocol development, data and sample collection, and testing . We also thank the RCGP practices for submitting samples. 

## Funding Information

The work at Imperial College was funded by a Medical Research Council UK Centre grant. We also acknowledge support from the NIH/NIGMS MIDAS programme, The Royal Society (CF), Research Councils UK (SC) and Bill and Melinda Gates Foundation (JTG, ACG,SC, CF, PW, NMF).  Imperial College (JT,NMF) and the Health Protection Agency (IMH, SL) acknowledge grant support from the European Union FP7 projects FluModCont and EMPERIE.  The views expressed in this paper are those of the authors and do not necessarily reflect those of the funders. 

## Author Contributions

ACG, JTG & NMF estimated the natural history parameters and probabilistic sources of infection. MB, SF, AJVH & BSC applied existing approaches to the UK data with input from WJE. MB, SF and BSC developed and implemented the first extension to the W&T method (Model 1) with input from AJVH, WJE and PW. ACG & NMF developed and implemented the probabilistic tree reconstruction model (Model 2) with input from JTG, SC and CF. JTG developed and implemented the full transmission models (Model 3 and the model based on POLYMOD contacts in the Supporting Information) with input from ACG, SC and NMF. IMH and IB estimated age-dependent susceptibility using travel data with input and steer from SL.  ACG, CAD, GK and RH undertook the household analysis with input from JT, HEJ, RP, BSC, and TG. JM, NP, AC, MZ, RP, PW and BSC developed the FF100 protocol with input from SC, ACG, IMH and NMF and others from the Health Protection Agency, Health Protection Scotland, Communicable Disease Surveillance Centre Northern Ireland and National Public Health Service Wales. JE developed and performed H1N1pdm assays. The data were collected under the responsibility of MC. AJVH and RP managed the data at the HPA. ACG, JTG, JT, HJ and TG managed the data at Imperial College. MW constructed the network pictures.  ACG, MB, JTG, SC, CAD, PW, NMF and BSC contributed to the initial drafting of the manuscript. All authors reviewed the final manuscript.  

## Competing Interests

The authors declare that no competing interests exist.

## Technical Appendix

A technical appendix is available on request from a.ghani@imperial.ac.uk. 
